# Supplementary material for: Detection and Control of Fusarium oxysporum from Soft Rot in Dendrobium officinale by Loop-Mediated Isothermal Amplification Assays
Source: Biology (Basel). 2021 Nov 5;10(11):1136. doi: 10.3390/biology10111136 (PMC8615024; doi:10.3390/biology10111136)
Supplement: Supplementary file 1 [file biology-10-01136-s001.zip › biology-1433237-supplementary.pdf]

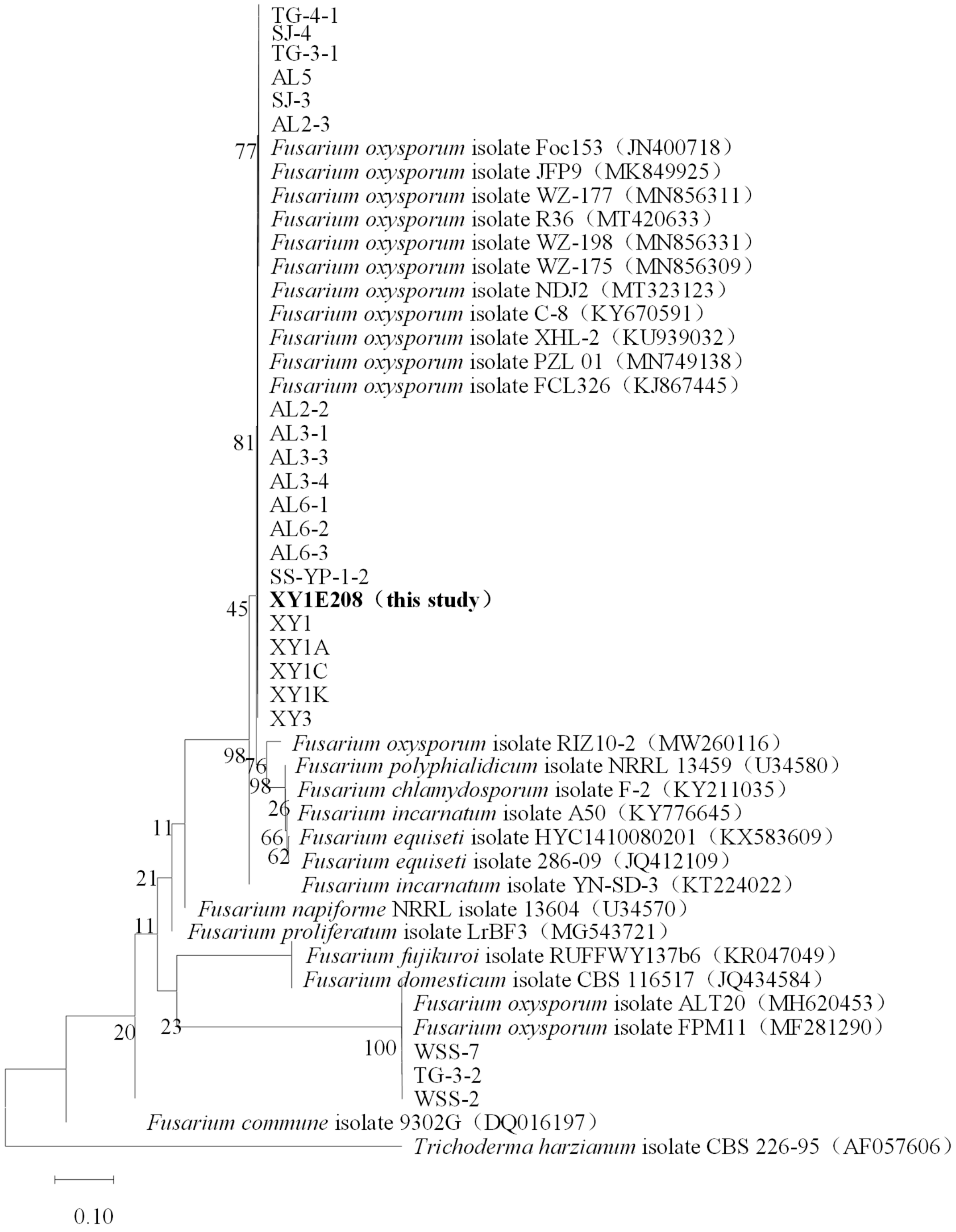

Figure S1 Phylogenetic tree for the 23 strains of *F. oxysporum* based on internal transcribed spacer (*ITS*) gene sequences

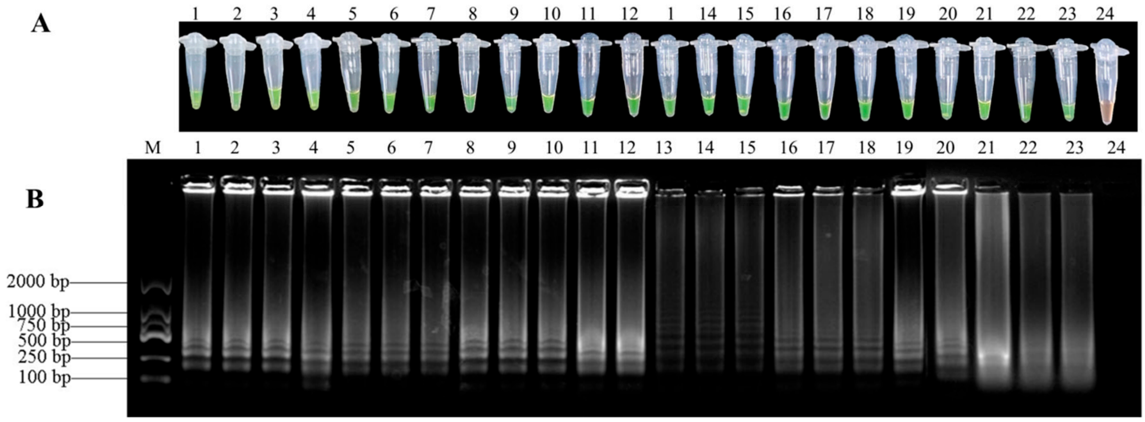

Figure S2. Sensitivity of LAMP and conventional PCR for detection *F. oxysporum* genomic DNA. A = Detection by LAMP with SYBR Green I staining; B = LAMP products analyzed by agarose gel electrophoresis. Lane M, Ds 2000 DNA molecular weight marker ladder; 1, XY1E208; 2, XY1; 3, XY1A; 4, XY1C; 5, XY1K; 6, XY3; 7, SJ-3; 8, SJ-4; 9,SS-YP-1-2; 10, TG-3-1; 11, TG-3-2; 12, TG-4-1; 13, WSS-2; 14, WSS-7.;15, AL2-2; 16, AL2-3; 17, AL3-1; 18, AL3-3; 19, AL3-4; 20, AL5; 21, AL6-1; 22, AL6-2; 23, AL6-3; 24, double distilled water as negative control.
